# Identification of cell proliferation, immune response and cell migration as critical pathways in a prognostic signature for HER2^+^:ERα^-^ breast cancer

**DOI:** 10.1371/journal.pone.0179223

**Published:** 2017-06-20

**Authors:** Jeffrey C. Liu, Miriam Zacksenhouse, Andrea Eisen, Sharon Nofech-Mozes, Eldad Zacksenhaus

**Affiliations:** 1 Division of Advanced Diagnostics, Toronto General Research Institute—University Health Network, Toronto, Ontario, Canada; 2 Brain-computer Interfaces for Rehabilitation Laboratory, Faculty of Mechanical Engineering, Technion—Israel Institute of Technology Haifa, Israel; 3 Sunnybrook Health Sciences Centre, University of Toronto; Toronto, Ontario, Canada; 4 Department of Medicine, University of Toronto, Toronto, Ontario, Canada; University of South Alabama Mitchell Cancer Institute, UNITED STATES

## Abstract

**Background:**

Multi-gene prognostic signatures derived from primary tumor biopsies can guide clinicians in designing an appropriate course of treatment. Identifying genes and pathways most essential to a signature performance may facilitate clinical application, provide insights into cancer progression, and uncover potentially new therapeutic targets. We previously developed a 17-gene prognostic signature (HTICS) for HER2^+^:ERα^-^ breast cancer patients, using genes that are differentially expressed in tumor initiating cells (TICs) versus non-TICs from MMTV-Her2/neu mammary tumors. Here we probed the pathways and genes that underlie the prognostic power of HTICS.

**Methods:**

We used Leave-One Out, Data Combination Test, Gene Set Enrichment Analysis (GSEA), Correlation and Substitution analyses together with Receiver Operating Characteristic (ROC) and Kaplan-Meier survival analysis to identify critical biological pathways within HTICS. Publically available cohorts with gene expression and clinical outcome were used to assess prognosis. NanoString technology was used to detect gene expression in formalin-fixed paraffin embedded (FFPE) tissues.

**Results:**

We show that three major biological pathways: cell proliferation, immune response, and cell migration, drive the prognostic power of HTICS, which is further tuned by Homeostatic and Glycan metabolic signalling. A 6-gene minimal Core that retained a significant prognostic power, albeit less than HTICS, also comprised the proliferation/immune/migration pathways. Finally, we developed NanoString probes that could detect expression of HTICS genes and their substitutions in FFPE samples.

**Conclusion:**

Our results demonstrate that the prognostic power of a signature is driven by the biological processes it monitors, identify cell proliferation, immune response and cell migration as critical pathways for HER2^+^:ERα^-^ cancer progression, and defines substitutes and Core genes that should facilitate clinical application of HTICS.

## Introduction

Breast cancer is a highly heterogeneous disease [1–6]. In the clinic, most (~65–70%) tumors are classified as estrogen receptor alpha positive (ERα^+^) that are treated with endocrine therapy [7]. About 20% is driven by amplification of the receptor tyrosine kinase HER2/ERBB2/NEU and either express ERα (HER2^+^:ERα^+^) or not (HER2^+^:ERα^-^). A third category comprises triple negative tumors (~10–15%), which do not express ERα, progesterone receptor or HER2. HER2^+^ patients are treated with chemotherapy plus HER2 antagonists such as trastuzumab, trastuzumab-emtansine, pertuzumab or lapatinib [8–13]. Without HER2-directed therapy, HER2^+^:ERα^-^ patients have the worst clinical outcome. Yet, some of these tumors do not progress to develop macro-metastases, and therefore surgical removal alone with local radiation or conventional therapy may suffice, at least as front-line therapy. Indeed, there is growing evidence that patients with small (T1), node negative HER2 positive tumors may profit from less aggressive therapies [14] [15]. In contrast, other tumors disseminate to form distal metastases that are virtually incurable, and should be treated most aggressively. Generating a prognostic signature from primary biopsies of these tumors may therefore help guide clinicians and patients as to the most appropriate treatment (Reviewed in Ref.[16]). Many such multi-gene based signatures have been developed for breast cancer and other malignancies [17–22]. Understanding the biology of these signatures and the basis for their prognostic power may provide further insight into processes that drive metastatic disease and mortality.

We previously reported on the development of a powerful prognostic signature for HER2^+^:ERα^-^ breast cancer patients [23]. Our strategy was based on the recognition that Tumor Initiating Cells (TICs), which sustain growth following transplantation into recipient mice, may express genes that drive metastatic dissemination, colonization and growth at distal sites [24]. We therefore developed a prognostic signature for HER2^+^:ERα^-^ patients using enriched TIC fraction from a mouse model for this subtype, MMTV-Her2/Neu [23, 25, 26]. Using both differentially up- and down- regulated genes between TIC-enriched and non-TIC fractions, we generated a 17-gene Her2-TIC-enriched signature (HTICS) that predicted clinical outcome on publicly available HER2^+^:ERα^-^ cohorts, but not HER2^+^:ERα^+^ patients [23]. When tested head-to-head, HTICS had superior prognostic power for HER2^+^:ERα^-^ patients than a HER2-Derived Prognostic Predictor (HDPP) [27], Stroma-Derived Prognostic Predictor (SDPP) [19], IGS [18], mammaPrint [28] or a proliferation signature [29]. Furthermore, in multivariate analysis, HTICS was independent of multiple clinical variables [23]. It predicted overall survival (OS) for HER2^+^:ERα^-^ patients with hazard ratio (HR) of 5.57 (P = 0.002), and metastatic free survival (MFS) with HR of 7.94 (P = 0.00084). Retrospective analysis on a small cohort of patients treated with trastuzumab (Herceptin) revealed that HTICS^+^ HER2^+^:ERα^-^ patients had a worse prognosis compared with HTICS^-^ patients but benefited from trastuzumab therapy, and therefore should be prioritized for anti-HER2 therapy [23]. In contrast, HTICS^-^ patients exhibited good prognosis and may benefit from withholding anti-HER2 therapy as a frontline treatment.

We hypothesized that a prognostic signature monitors the combinatory effect of several biological pathways, and that understanding these pathways may shed light into the biology behind aggressive forms of a particular cancer subtype. The identification of a minimal “core” set of genes may also help define essential pathways. From a clinical perspective, a minimal set of genes may also be useful in generating immunohistochemical (IHC)-based prognosis. In addition, targeting these core genes or pathways may be therapeutically beneficial. Finally, it is important to identify substitutes that can be used to replace specific genes in a signature. If such substitutions are derived from the same functional pathways as the original signature genes, one can further establish the importance of the pathway. Such substitutions are also useful because most signatures are developed using RNA microarray data derived from fresh biopsies, whereas clinical biopsies are routinely processed as formalin-fixed paraffin embedded (FFPE) tissues, necessitating other modes of transcript detection such as NanoString technology [30]. Substituting genes may be required to compensate for limited technical performance of certain probes when a molecular signature acquired from frozen tissues is applied to another platform such as FFPE. This is especially critical for small signatures in which the contribution of each gene is highly important.

In the present study, we describe the identification of biological pathways monitored by HTICS and demonstrate that most genes capable of substituting for this signature share the same pathways. Expression of HTICS genes and their substitutes could be reliably detected in FFPE samples from breast cancer patients using NanoString technology, thus extending the clinical utility of HTICS. Through gene combination studies we have identified a core of 6 genes in HTICS and validated the effectiveness of this core using an independent RNA-Seq platform.

Together, our results demonstrate that a prognostic signature reflects critical biological pathways in a particular breast cancer subtype that dictate patient outcome. Specifically, we found that the prognostic power of HTICS lies in its ability to track cell proliferation, migration and immune-response pathways, which are critical for HER2^+^:ERα^-^ tumorigenesis. Our analysis also identifies a core and substituting genes that would facilitate clinical implementation of HTICS. The critical role of the immune response, even within the 6 gene Core, for the prognostic power of HER2^+^:ERα^-^ patients is particularly important given the accumulative evidence linking the immune system to the therapeutic effects of trastuzumab [31].

## Materials and methods

### Research ethics board (REB)

Experimental protocols with human breast cancer samples were approved by Sunnybrook Health Sciences Centre REB (PIN 418–2011), and were carried out in accordance with the approved guidelines.

### Amplicon analysis

HER2 IHC data from GSEs 24185, 22358, 25066, 2603, 2034, 5460, 21653, 26639, 19697, 17907, 16446, & 20194 (**S1 Table**) were pooled together and compared to expression levels of 5 HER2 gene amplicon (Her2/Erbb2, Stard3, Perld1, Grb7, C17orf37). Normalized gene expression values were used to determine fold changes against the median across the samples. Samples with greater than 2-fold elevation in 3 of 5 genes in the amplicon were considered HER2^+^ (**S1 Table**). We note that at this cutoff (2-fold) we achieve the best balance of Positive Predictive Value (PPV) = 80.7% and sensitivity = 69.5%. Higher cutoff further enhance the PPV (>90%) at the expanse of sensitivity (total number of IHC HER2+ samples captured). ROC analysis was performed comparing HER2^+^ status as determined by amplicon genes with IHC.

### Score for Signature Match (SSM) Classifier

For the comparison of effectiveness of our signatures, we used the Score for Signature Match (SSM) classifier to determine the status (signature match or no match) of each sample in the cohort [23]. In short, the SSM classifier takes into consideration 2 major aspects of the signature genes for calculation: 1) the rank of each gene among the samples in the cohort (X_n_). This is measured by median-centering the log2 transformed gene expression value, where a positive value is given to X_n_ if the gene is expressed at levels above the median of the cohort and a negative value if below; 2) the expected direction (up or down) and weight of the gene (I_n_). We gave every gene equal weight and therefore I_n_ = +1 for Up-regulated signature genes and I_n_ = -1 for Down-regulated signature genes. The score for each gene is the product of I_n_ and X_n_ (I_n_ x X_n_ / |X_n_|). The formula for SSM is:

$$SSM=\frac{\sum \left(\frac{{I_{n}X}_{n}}{\left|X_{n}\right|}\right)}{\sum \left(\left|I_{n}\right|\right)}$$

The formula can be simplified as: Sum of Scores for Every Gene in Signature / Total Weight. If a gene is supposed to be up-regulated (I_n_ = 1), a sample with positive expression above median (X_n_ > 0) will be I_n_X_n_/|X_n_| = 1. If a sample is below median expression (X_n_ < 0) for the same gene, I_n_X_n_/|X_n_| = -1 leading to deduction of SSM score. Samples with SSM ≥0 were considered a match for the signature and used to compare with all other samples with SSM <0.

### Receiver Operating Characteristic (ROC) analysis

The ROC curve represents the tradeoff between True-Positive (correct match) and False-Positive (incorrect match) rates as the threshold level of the classifier varies. The Area Under the ROC Curve (AUC) provides a standard measure of the discrimination power of the classifier, with 1/2 indicating no discrimination power and 1 indicating perfect discrimination. We used ROC and AUC to assess and compare the discrimination/prognostic power of the different signatures/combinations. ROC analysis was performed using algorithms developed by John Eng from the Russell H. Morgan Department of Radiology and Radiological Science at Johns Hopkins University School of Medicine (http://www.rad.jhmi.edu/jeng/javarad/roc/JROCFITi.html). In brief, the score of each sample for a given signature/combination was calculated by Score for Signature Match (SSM) algorithm and compared to a threshold to determine the match/no-match groups. The match/no-match class was compared with the survival events in the datasets. The survival events of patients were divided into 4 categories to reflect the degree of cancer aggressiveness: 1) disease-free patients after 36 months, the least aggressive; 2) disease-free patients within 36 months, not aggressive; 3) patients’ disease progressed after 36 months, aggressive; 4) patients’ disease progressed within 36 months, most aggressive.

### Human breast cancer patient samples and FFPE RNA extraction

Formalin-fixed paraffin-embedded breast cancer samples from the Department of Anatomic Pathology at Sunnybrook Hospital, Toronto, were used to generate 5-μm thick tissue section slides. Tumors were classified to HER2^-^:ERα^+^, HER2^+^:ERα^-^, HER2^+^:ERα^+^ and triple-negative using immunohistochemistry and FISH according to standard methodology. Sections were macrodissected to enrich the sample for invasive component, and de-waxed through sequential 10-minutes washes in xylene (3x), 100%, 90%, 75%, 50%, 30% Ethanol, and PBS. The de-waxed samples were then scrapped off by surgical scalpels and placed in 1.5 ml eppendorf tubes. RNA was extracted using High Pure FFPE RNA Micro Kit (Roche, cat# 04823125001).

### Human peripheral blood mononuclear cell, breast cancer cell lines and RNA extraction

Frozen human peripheral blood mononuclear cell (PBMC) were gifts from Dr. Shannon Dunn in the Department of Immunology, University of Toronto. MDA157, MDA231, MDA361, MDA436, MDA468, BT549, CAMA1, JIMT1. HCC3153 and HCC38 cells were cultured as described [22, 32]. The cells were plated in 10 cm plates and harvested at 80% confluence. RNA was extracted from cells using RNeasy Mini Kit (Qiagen, cat#74104).

### Datasets with clinical outcomes

Published datasets were downloaded from GEO datasets at NCBI, NIH (http://www.ncbi.nlm.nih.gov/gds/). Datasets analyzed by Affymetrix microarray without Herceptin treatment (a potential cofounding factor) but with clinical outcomes, included all breast cancer subtypes. They were used for Kaplan-Meier, ROC and random signature analyses (**S2 Table**). Datasets with Overall survival (OS) outcomes were: GSEs 16446 and 20685. Datasets with Metastasis-Free Survival (MFS) outcomes were: GSEs 2034, 2603, 5327, 6532, 11121, and 25066 (**S3 Table**).

### Pathway analysis

**Fig 1:**
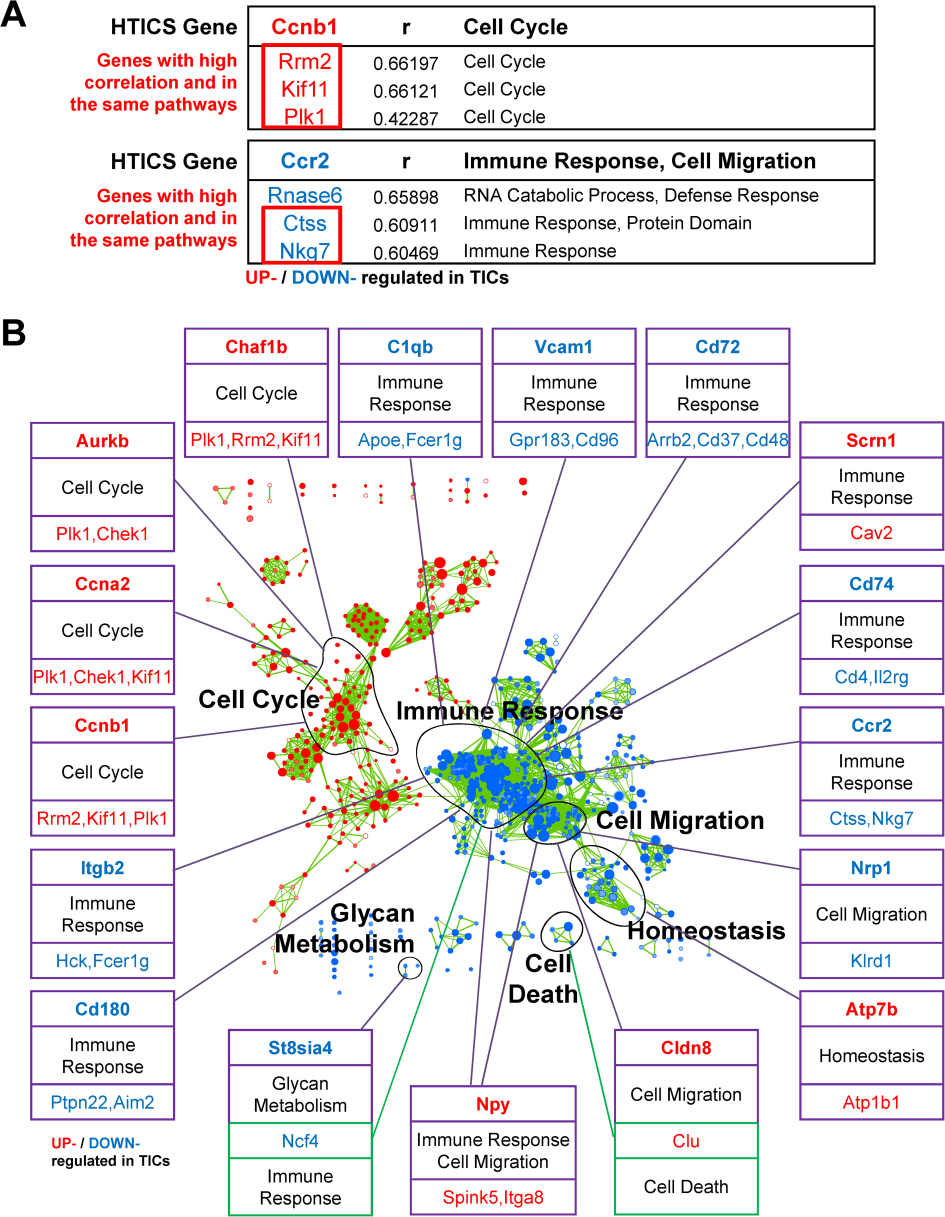
Identification of Substitutes for HTICS Genes. (A) Examples of replacement genes and corresponding functional pathways for Ccnb1 and Ccr2. (B) Replacement genes and corresponding pathways in relation to Enrichment maps for all HTICS genes. Note that 15/17 substitutes are from the same pathways as the original HTICS genes.

Microarray analysis with mouse tumor models was carried out using Affymetrix Mouse Gene 1.0 ST with 500 ng of total RNA isolated by double Trizol extractions (Centre for Applied Genomics, Hospital for Sick Children, Toronto). Microarray data were normalized using RMA method via Partek software and log2-transformed gene expression values were obtained. The data were analyzed by GSEA using paired t-test comparing gene expression values in the TIC and CD24- fractions, and parameters set to 2,000 gene-set permutations, gene-sets size between 15 and 500 [23]. The data is deposited in GEO Datasets with identifiers GSE29590 and GSE29616. An enrichment map (version 1.1 of Enrichment Map software) was generated using enriched gene-sets with a nominal p-value < 0.005, FDR < 1% and the overlap coefficient set to 0.5 [33, 34]. The databases included in the GSEA analyses were the Gene Ontology (GO), KEGG, PFAM, BIOCARTA and NCI databases. GO, PFAM and KEGG annotations were downloaded from Bioconductor (org.Mm.eg.db version 2.4.6, GO.db version 2.4.5, KEGG.db version 2.4.5). NCI annotations were downloaded from NCI website (http://pid.nci.nih.gov/, 2010-11-08) and BioCarta annotations downloaded from WhichGenes (2010-03-26). Only highly significant gene-sets (p < 0.005; FDR < 1%) are shown as nodes in the enrichment map. When two nodes have high degree of overlap in genes (overlap coefficient > 0.5), they are connected by a green line (edge). The major pathways labeled in Fig 1B are clusters of these significant gene-sets (nodes) by the Markov Cluster Algorithm.

### NanoString analysis

Total RNA from samples were diluted to 20 ng/μl concentration and at least 50 ng of RNA was sent for NanoString analysis (**S4 Table**). NanoString probes were designed according to Ref sequences with the following ID: AURKB (NM_004217.2); CCNA2 (NM_001237.2); SCRN1 (NM_001145513.1); NPY (NM_000905.2); ATP7B (NM_000053.2); CHAF1B (NM_005441.2); CCNB1 (NM_031966.2); CLDN8 (NM_199328.2); NRP1 (NM_001024629.2); CCR2 (NM_001123041.2); C1QB (NM_000491.3); CD74 (NM_001025159.1); VCAM1 (NM_001078.3); CD180 (NM_005582.2); ITGB2 (NM_000211.2); CD72 (NM_001782.2); ST8SIA4 (NM_005668.4); Spink5 (NM_006846); Ctss (NM_004079); Aim2 (NM_004833); CD48 (NM_001778). The genes were normalized using the following controls: C4ORF8 (NM_003704.3); CAPZB (NM_001206540.1); EIF3A (NM_003750.2); RTN3 (NM_201429.1). The samples were processed and read by nCounter Analysis System from NanoString through UHN facilities (http://www.uhnresearch.ca/facilities/microarray.htm).

### Algorithms for random sets and combination analysis

To determine the probability that signatures/combinations could be the result of random chance, we generated 1000 random signatures with the same composition (i.e. same number of up- and down-regulated genes) and estimated their HRs using a formula provided by Kleinbaum and Klein (Chapter 3, Survival Analysis, 2005) with the same patients as indicated [35]. Algorithms for random sets as well as for combination analysis both written in R are available upon request.

### Additional statistical analysis

Parametric paired samples were analyzed by student t-test. Kaplan-Meier Survival analysis was performed using the PAST program (P.D. Ryan and Ø. Hammer, University of Oslo) and p-value was calculated using the Wilcoxon method. Hazard ratios were calculated by Cox Proportional Hazards Survival Regression. Pearson correlation studies were also performed using the PAST program. Differences between values were considered statistically significant at p< 0.05.

## Results

### ROC analysis of HER2 amplicon versus IHC for HER2^+^ breast cancer classification

As a prelude to the forthcoming analysis, we assessed the assay we employ to call HER2^+^ breast cancer samples. This assay, developed by Staaf *et al*., measures expression levels of 5 genes around the HER2 amplicon (Her2/Erbb2, Stard3, Perld1, Grb7, C17orf37) directly from microarray data to predict HER2 status [23, 27]. The advantage of this approach is that the status of HER2, determined by IHC or FISH, is only available for a fraction of cohorts with mRNA profiling and clinical outcome. Moreover, methods and standardization for HER2 expression vary across clinical labs. To assess whether the amplicon strategy is as efficient as IHC in identifying HER2^+^ breast cancer samples, we performed Receiver Operating Characteristic (ROC) analysis. This analysis calculates the discriminatory power of a classifier by describing the trade-off between True-Positives (correctly predicting HER2 status from amplicon expression as positive) and False-Positives (incorrectly predicting HER2 status as positive). For this analysis, we combined 11 cohorts containing a total of 1833 breast cancer samples with microarray and IHC data (although most have no clinical outcome, **S1 Table**). HER2 amplicon status (HER2 positive or negative for 5 amplicon gene expression) was then compared to HER2 IHC classification (positive or negative IHC; details in Method and Materials, S1 Table). Fisher’s exact test demonstrated a significant detection of IHC HER2^+^ samples by HER2 amplicon genes with 96% specificity (**S1 Table**). The HER2 amplicon accurately predicted HER2 IHC status of breast cancer patients with an Area Under Curve (AUC) value of 0.90 (**S1A Fig**). Together, the two methods have a Concordance Rate of 90.9% and Cohens Kappa coefficient of 0.69. Thus, in agreement with a recent publication [36], microarray amplicon analysis is a reliable surrogate for HER2 status as determined by IHC, and can be used to improve consistency across datasets.

We previously demonstrated that HTICS is particularly effective in prognostication of HER2^+^:ERα^-^ patients. This specificity is also seen by ROC analysis of metastasis-free (MFS) and overall (OS) survival data, having AUCs of 0.80 and 0.69, respectively, for HER2^+^:ERα^-^ patients. In contrast, AUCs for all HER2^+^ patients (MFS: 0.75; OS: 0.62) or HER2^+^:ERα^+^ patients (MFS: 0.72; OS: 0.53) were much reduced (**S1B Fig**).

### Pathway analysis of HTICS

We generated HTICS by comparing gene expression profiles of enriched fractions of tumor initiating cells (TICs) versus non-TICs from mammary tumors isolated from a mouse model of HER2 breast cancer (MMTV-Her2/Neu) [23]. Originally 283 significantly and differentially expressed genes were identified and through training, described in reference [23], were narrowed down to 17 critical genes with strong prognostic power (as shown with MFS data, **S1C Fig**). We identified enriched biological pathways in TIC relative to non-TIC fractions using Gene-set-enrichment analysis (GSEA) and Enrichment Map (EM) via Cytoscape [23] (**Table 1**). This analysis suggests that HTICS monitors several pathways: Cell Cycle (Aurkb, Ccna2, Chaf1b, and Ccnb1, all up-regulated in TICs), Cell Migration (Npy, Cldn8, up-regulated; Nrp1, down-regulated), and Immune Response (Scrn1, up-regulated; C1qb, CD74, Ccr2, Itgb2, Vcam1, CD180, CD72, down-regulated). In addition, HTICS includes one Glycan Metabolism gene St8sia4 (down-regulated), involved in polysialic acid synthesis, and one homeostasis gene, Atp7b (up-regulated), required for efflux of copper out of the cells.

**Table 1:** HTICS genes and corresponding pathways as determined by GSEA.

HTICS	Entrez ID	Name	Major Pathway
**Aurkb**	20877	Aurora Kinase B	**Cell Cycle**
**Ccna2**	12428	Cyclin A2	
**Chaf1b**	110749	Chromatin assembly factor 1, subunit B	
**Ccnb1**	268697	Cyclin B1	
**Scrn1**	69938	Secernin 1	**Immune Response**
**C1qb**	12260	Complement component 1, q subcomponent binding protein	
**CD74**	16149	CD74 molecule	
**Ccr2**	12772	Chemokine (C-C motif) receptor 2	
**Itgb2**	16414	Integrin, beta 2	
**Vcam1**	22329	Vascular cell adhesion molecule 1	
**CD180**	17079	CD180 molecule	
**CD72**	12517	CD72 molecule	
**Npy**	109648	Neuropeptide Y	**Cell Migration**
**Cldn8**	54420	Claudin 8	
**Nrp1**	18186	Neuropilin 1	
**Atp7b**	11979	ATPase, Cu++ transporting, beta polypeptide	**Homeostasis**
**St8sia4**	20452	ST8 alpha-N-acetyl-neuraminide alpha-2,8-sialyltransferase 4	**Glycan Metabolism**

UP- / DOWN- regulated in TICs

### Leave-one-out analysis of HTICS pathways

To assess the contribution of each pathway as a whole, we deleted each at a time and assessed the prognostic power (as measured by Hazard Ratio and AUC) of the remaining genes, using combined data from 12 publically available datasets with microarray gene expression profiles and clinical outcome [23]. We found that leaving out each of the five pathways diminished the prognostic power of HTICS, albeit to different extents, indicating that all pathways contribute to its full prognostic power (**Table 2**). Most critical pathways were Cell cycle and Immune response, followed by Glycan metabolism, Homeostasis and Cell migration. Notably, by this analysis Glycan metabolism and Homeostasis (one gene each) are more critical than Cell migration (3 genes). However, the detailed analysis below reveals that Cell migration is more important than Glycan metabolism and Homeostasis, and that the three main pathways (Cell cycle, Immune response, Cell migration) are most significant, yet genes from all five pathways contribute to the prognostic capability of HTICS.

**Table 2:** Leave-one out analysis assessing the effect of each pathway on HTICS prognostic power.

Pathway Removed	MFS	
	HR	AUC
**None**	**7.94**	**0.80**
**Cell Cycle**	**2.86**	**0.68**
Cell Migration	7.17	0.78
**Immunue Resp.**	**1.84**	**0.72**
Homeostasis	5.45	0.71
Glycan Metab.	5.13	0.71

### Identification of substitutes for HTICS genes

Having demonstrated that HTICS monitors the activity of specific signaling pathways in HER2^+^ breast cancer, we next asked whether genes in the same, rather than all other, pathways would preferentially substitute for HTICS genes. To identify such potential replacements, we initially performed correlation studies on each of the additional 266 TIC genes (i.e. from the original 283 differentially regulated genes in TICs, omitting the 17 HTICS genes) that are significantly regulated in enriched-TICs vs. non-TICs in MMTV-Her2/Neu tumors [23]. Specifically, for each of the 17 HTICS genes, a correlation analysis was performed to determine which of the 266 differentially regulated genes had the most similar expression pattern. Remarkably, 15 of the 17 top correlated genes shared the same pathways as the HTICS genes they could replace (**Fig 1A**). The only 2 exceptions were Cldn8 (Cell Migration), which was best replaced by Clu (Cell Death) and St8sia4 (Glycan Metabolism), which was best substituted by Ncf4 (Immune Response) (**Fig 1B**). Of the 15 genes with potential substitutes in the same pathway, two had the highest correlation with genes from different pathways: CCR2 (Immune Response) could be substituted by Rnase6 (RNA Catabolic Process), and Scrn1 (Immune Response) could be replaced by Mphosph6 (not associated with any identified pathway) (**Fig 1; S2 Fig**). Our observation that for most HTICS genes (15/17), the best substitutes share similar functional pathways underscores the importance of these pathways in predicting disease progression and survival of HER2^+^:ERα^-^ patients.

### Development of Nanostring probes for HTICS and detection in FFPE samples

**Fig 2:**
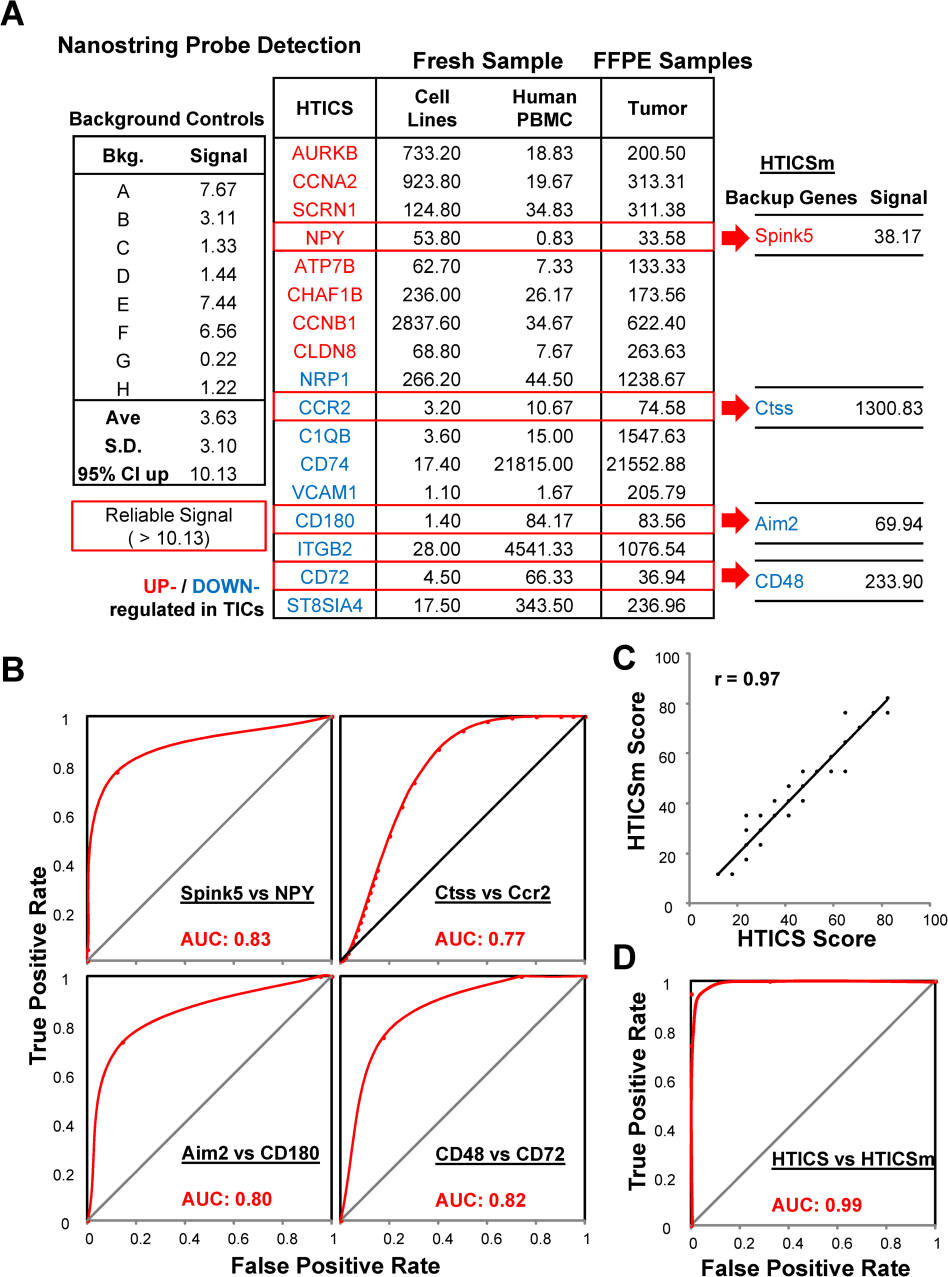
Analysis of substitute genes versus HTICS. (A) Reliable detection of gene expression of HTICS and substitutes in cell lines, PBMC and FFPE tumor samples using NanoString probes. (B) ROC analysis of individual substitute gene in comparison with the original HTICS gene. (C) High degree of correlation (r = 0.97) between HTICS and HTICSm. (D) ROC analysis demonstrating near perfect agreement between HTICS and HTICSm in segregating patients with AUC of 0.99.

To assess the clinical utility of HTICS, we designed Nanostring probes for the signature genes. As noted, HTICS was generated using RNA microarray data acquired from fresh/frozen tumor biopsies. However, in a clinical setting, the routine platform for detecting gene expression is formalin-fixed paraffin-embedded (FFPE) samples. RNA quality from FFPE tissues is poor, and differences in sample preparation may introduce artifacts that can render detection of some genes unreliable. Knowledge of signature pathways and substitutes could therefore enable the design of clinical prognostic kits with built-in backup genes and controls to expand the clinical utility of HTICS. To test this strategy, we employed NanoString technology, which can faithfully detect RNA expression from partially degraded and contaminated RNA because it does not use an amplification step [30]. To detect the same spliced RNA transcripts, we designed the 17 NanoString probes for the same regions in the mRNA as in the microarray platform used to generate HTICS. These NanoString probes detected expression of all 17 HTICS genes from FFPE tumor samples (**Fig 2A**). By comparing signals generated from human breast cancer cell lines and peripheral blood mononuclear cells (PBMC), we could determine the tissue from which signals of each HTICS gene originated. For example, Cldn8 expression was predominantly detected in tumor epithelial cells, with very low signal in PBMC. In contrast, CD74 was exclusively detected in PBMC (**Fig 2A**). Vcam1 and C1qb were undetectable in both tumor epithelia and PBMC but readily detected in whole tumors, suggesting expression in other cell lineages/biological contexts such as endothelium/ coagulation (**Fig 2A**).

### Prognostic power of HTICS substitutes

To determine the ability of the potential replacements to predict clinical outcome, we designed NanoString probes for four substitutes (Spink5, Ctss, Aim2 and CD48). These substitutes were selected because the original HTICS genes (Npy, Ccr2, CD180 and CD72) gave low expression signals in FFPE sections (<100). The replacement genes exhibited comparable or even better expression (**Fig 2A)**. Moreover, ROC analysis of the pattern of gene expression of each substitute compared to the original HTICS gene in all the 79 MFS patients revealed high AUCs of 0.83, 0.77, 0.80 and 0.82 when using the Spink5, Ctss, Aim2 or CD48 substitutes, respectively (**Fig 2A and 2B**). A modified HTICS signature (HTICSm) with all 4 genes substituted gave nearly identical score in predicting outcome as HTICS, with a correlation of 0.97 (**Fig 2C**). HTICSm and HTICS also segregated patients into the same risk groups with AUC of 0.99 via ROC analysis (**Fig 2D**). Thus, these substitutes should produce consistent results by the NanoString assay, and thus, may be included in a prognostic kit for HER2^+^:ERα^-^ patients.

**Fig 3:**
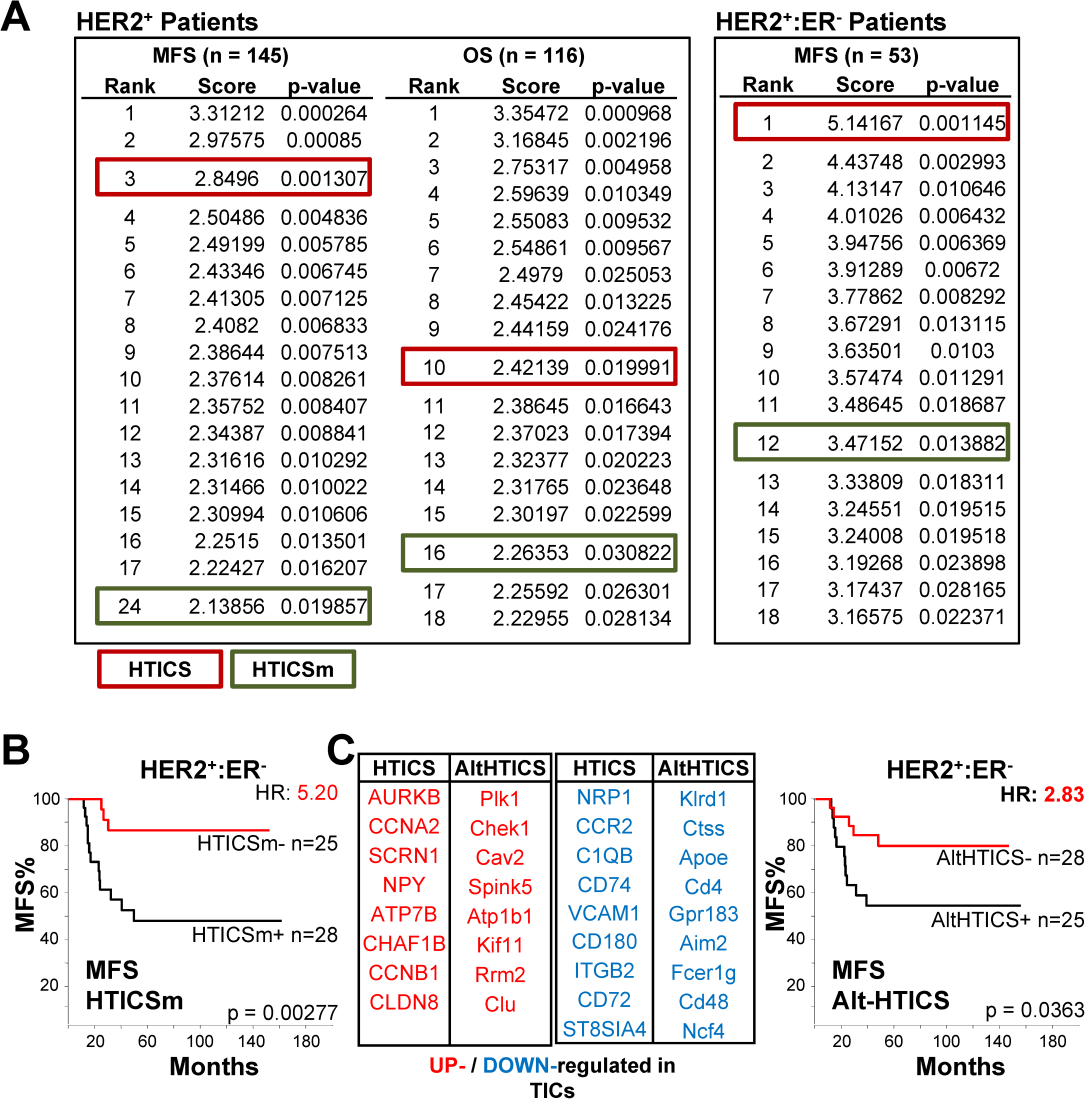
Comparison to 1000 random signatures and survival analysis of HTICSm and AltHTICS. (A) HTICS and HTICSm tested against 1000 randomly generated signatures of equal numbers of up- and down-regulated genes. (B) Kaplan-Meier analysis with HTICSm on MFS cohorts. (C) Prognostic power of AltHTICS with 17 gene substitutes, on MFS cohorts.

To assess the prognostic power of HTICSm and HTICS in a non-biased way, we generated 1000 randomly selected sets of 17 genes and compared their prediction power to those of HTICS and HTICSm [35]. Both signatures were highly prognostic, ranking within the top 25 for HER2^+^ patients and top 12 for HER2^+^:ERα^-^ patients (**Fig 3A**). Yet, HTICS performed consistently better than HTICSm and was ranked #1 for HER2^+^:ERα^-^ patients (**Fig 3A**). It had the highest prognostic power with hazard ratio (HR) of 7.94 for MFS and 5.57 for OS, as compared to HTICSm with HR of 5.20 for MFS, and HR of 4.01 for OS (**Fig 3B**).

When replacing all 17 HTICS genes with the substitutes, the alternative signature (AltHTICS) still made weak but significant prognosis for HER2^+^:ERα^-^ patients with HR of 2.83 for MFS (**Fig 3C**). In those cases where the highest correlated genes were from different pathways (Rnase6 for CCR2; Mphosph6 for Scrn1), substitution with other-pathway genes resulted in inconsistent (Rnase6 in AltHTICSb), or insignificant (Mphosph6 in AltHTICSc) prognosis **S2 Fig**). Thus, while the substitutes are capable of significant prognostication, likely because they monitor similar pathways, they are not as effective as the original HTICS.

### Identifying HTICS core genes

To define a core gene set that is most critical within HTICS, we developed an algorithm in R to test the prognostic power of all 131,071 possible combinations of genes in HTICS using both MFS and OS cohorts. Of these, 15,417 gene combinations were found to produce significant prognosis (P<0.05) with an average signature length of 9.8 genes.

**Fig 4:**
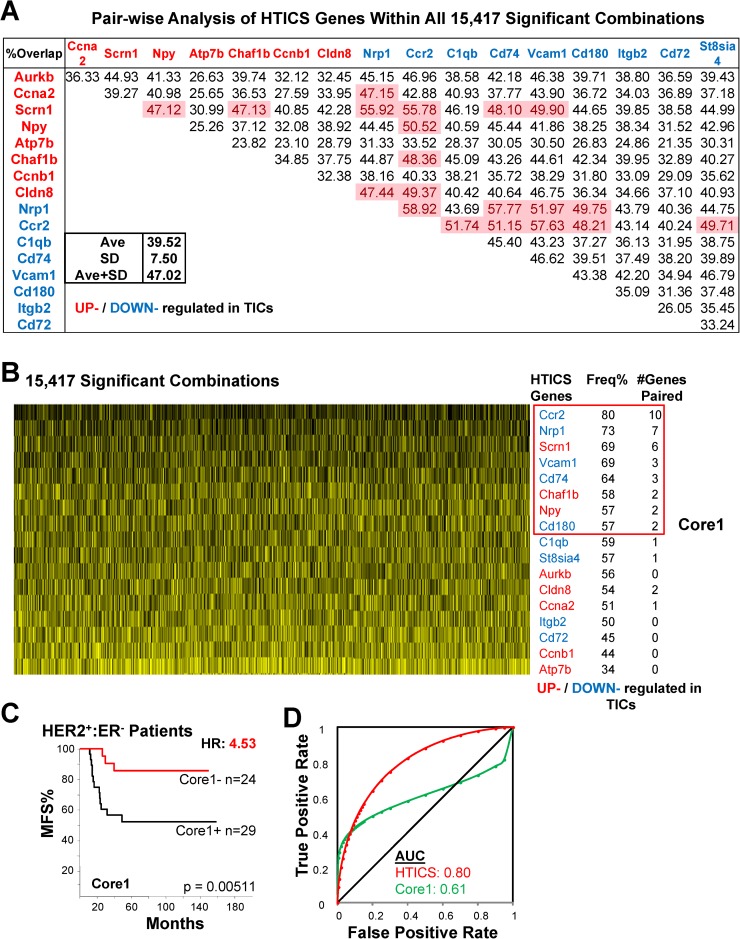
Identification of Core 1 by analysis of all 15,417 HTICS gene combinations. (A) Pair-wise analysis using 15,417 combinations that generated significant prognosis in both MFS and OS datasets. The frequency of combinations containing both genes in a pair is shown; pairs with high frequencies are highlighted in red. (B) Frequency of appearance of each HTICS gene in the 15,417 significant combinations and identification of Core 1. Vertical black lines indicate a gene is present in each combination; yellow lines point to its absence. Frequency and number of HTICS genes paired are listed. (C) Kaplan-Meier analysis of Core1 on MFS cohorts. (D) ROC analysis of Core1.

**Fig 5:**
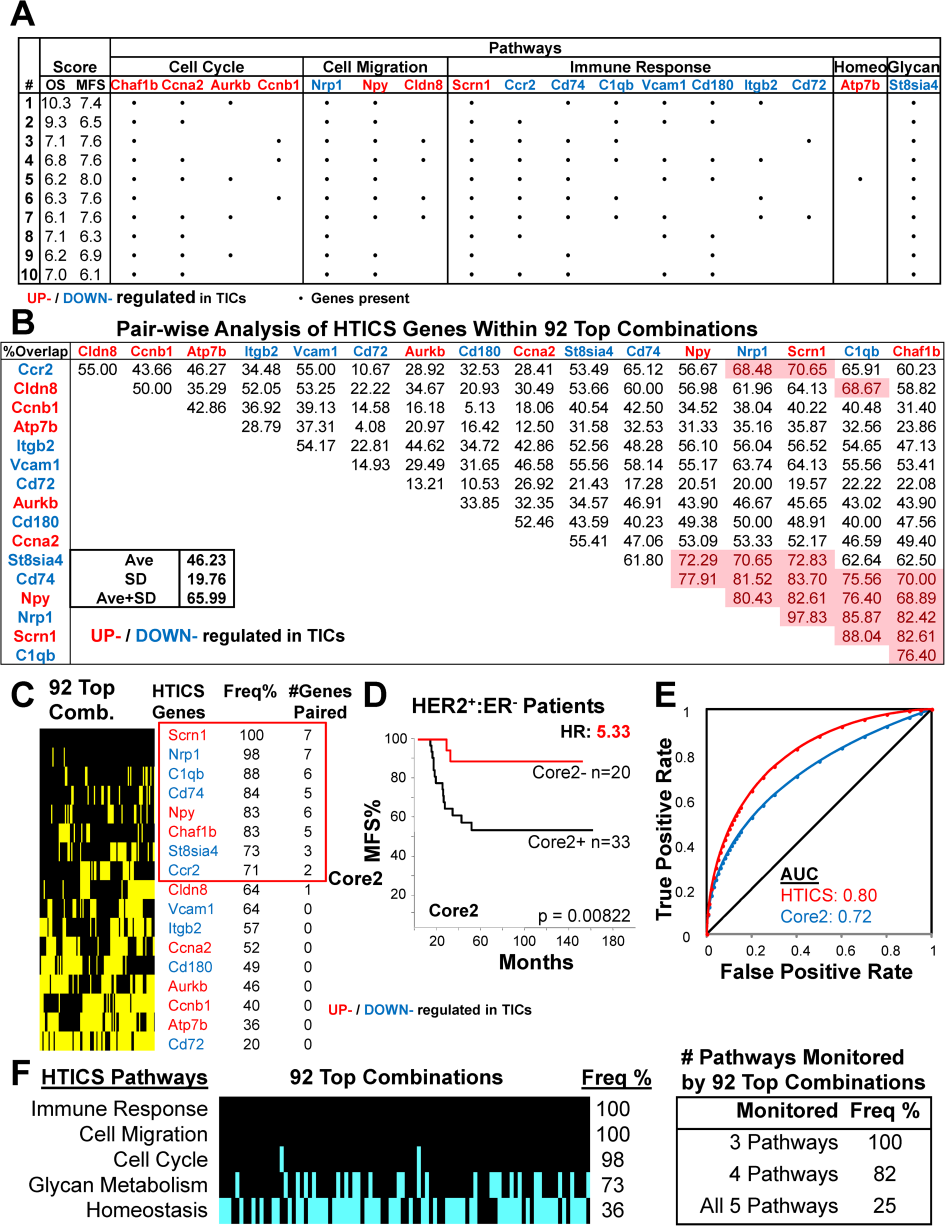
Identification of Core 2 by analysis of Top 92 HTICS gene combinations. (A) Top 10 gene combinations (of 92) with superior prognostic power. • denotes the presence of a gene in the combination. (B) Pair-wise analysis using the 92 superior combinations. Genes that paired with at least 2 HTICS genes with >1 standard deviation above average are highlighted in red. (C) Frequency of appearance of each HTICS gene in the 92 top combinations. Vertical black lines indicate the gene is present in each combination; yellow lines indicate its absence. Core2 includes genes with higher than median frequency that paired with at least 2 genes. (D) Kaplan-Meier analysis of Core2 on MFS cohorts. (E) ROC analysis of Core2. (F) Frequency of the 5 HTICS pathways in the 92 superior combinations. Black lines indicate a pathway is present in each combination; cyan lines indicate its absence. All combinations monitored at least 3 pathways as indicated.

We used two criteria to select for gene combinations with prognostic power. First, we asked how frequently a gene appears with other genes within the 15,417 significant prognostic signatures. For this, we performed a pair-wise analysis for each of the 17 HTICS genes to determine the percentage of overlaps with the other 16 genes. The average % of overlap was 39.52% with standard deviation (SD) of 7.5%. If the percentage overlap of a pair of genes was greater than 1 SD above the average, i.e. 47.02%, the two genes were considered to require each other for prognosis (**Fig 4A**). To further increase the stringency of selecting critical genes, we only considered those that paired with two or more other genes. 8 such genes highlighted in pink in Fig 5A were identified: Ccr2, Nrp1, Scrn1, Vcam1, CD74, Chaf1b, Npy and CD180, and were designated Core1 (**Fig 4B**). Core1 gave a HR of 4.53 for HER2^+^:ERα^-^ patients (**Fig 4C**), and AUC of 0.61 with high false positive rate by ROC analysis (**Fig 4D**).

Interestingly, of the 15,417 gene combinations with significant prognosis, 92 (with average length of 11.1 genes), gave similar or even better score than HTICS. Composition of the top 10 signatures and corresponding HRs in OS and MFS cohorts are depicted in **Fig 5A**. The 92 signatures maintained the cell migration, cell cycle and immune-response pathways; a relatively high percentage of them also included the glycan metabolism gene St8sia4, but not the homeostasis gene, Atp7b (**Fig 5A and 5B**). At least 3 of the 5 HTICS pathways were found in 100% of these 92 highly prognostic sub-signatures (**Fig 5C**). Whether these HTICS-derived sub-signatures are indeed superior to HTICS or whether they represent over-fitting awaits analysis of additional/new cohorts. Here, we used these 92 top combinations in a second approach to identify core genes for HTICS employing the pair-wise analysis described above (**Fig 5D**). This analysis also resulted in a core of 8 genes (designated Core2): Scrn1, Nrp1, C1qb, CD74, Npy, Chaf1b, St8sia4 and Ccr2 (**Fig 5B**). Core2 was a better predictor than Core1 with HR of 5.33 for HER2^+^:ERα^-^ patients and AUC of 0.72 by ROC analysis (**Fig 5E and 5F**).

**Fig 6:**
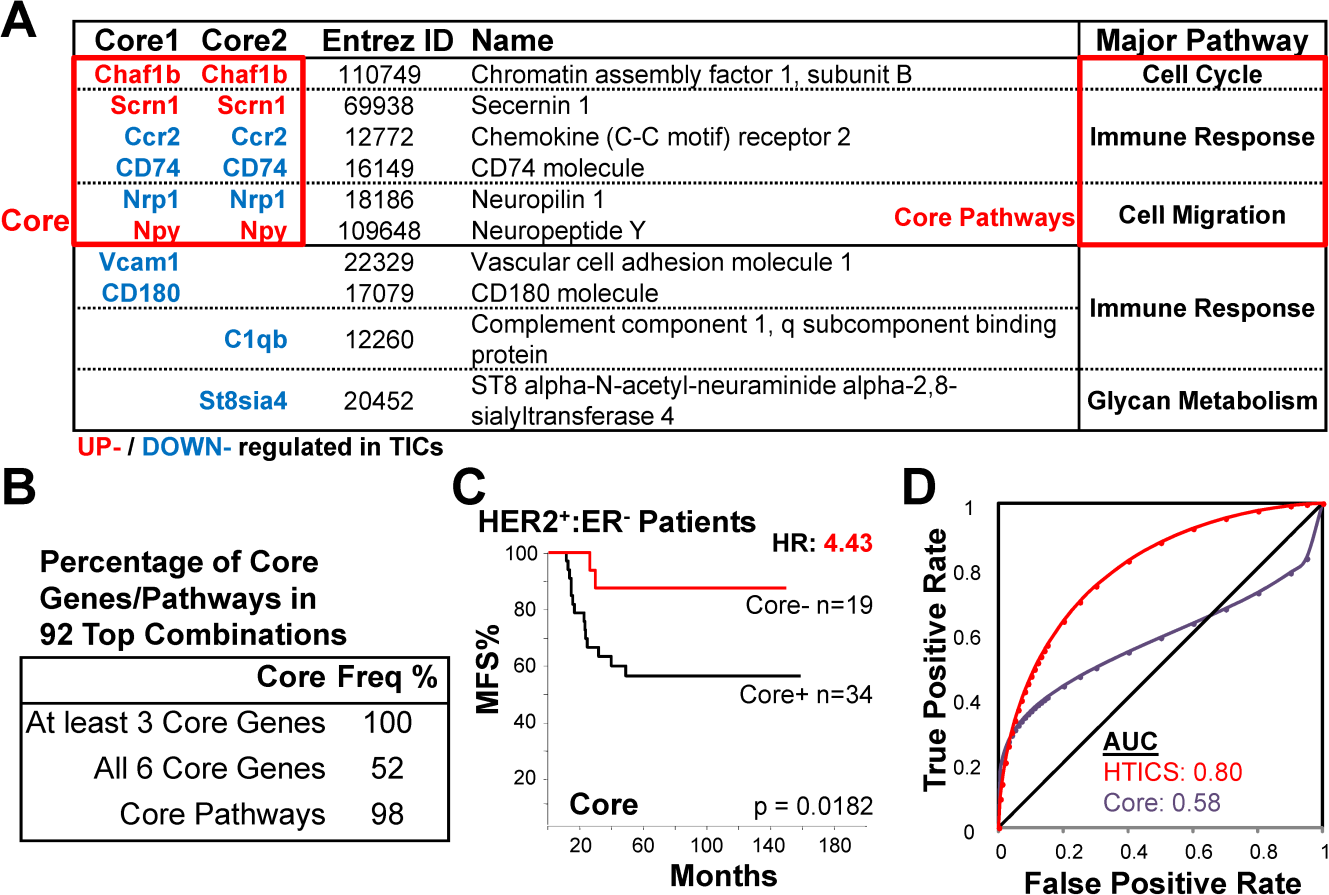
Identification of 6 common Core genes for HTICS. (A) Comparison between Core1 and Core2 and identification of a common 6 gene Core. (B) Frequency of the 6 Core genes and 3 essential pathways in the 92 good combinations. (C) Kaplan-Meier analysis of Core on MFS cohorts. (D) ROC analysis of Core.

Comparison of Core1 and Core2 revealed 6 common genes, 3 of which (Chaf1b, Scrn1, Npy) were up-regulated and 3 (Ccr2, CD74, Nrp1) down-regulated in enriched TICs (**Fig 6A**). The 6-gene “Core” monitored Cell Cycle, Cell Migration and Immune Response pathways but excluded the two other minor pathways/genes (Atp7b, St8sia4; **Fig 6A and 6B**). It significantly predicted survival of HER2^+^:ERα^-^ patients with HR of 4.43 (**Fig 6C**), but exhibited a relatively weak AUC of 0.58 by ROC analysis due to high rate of false positives (**Fig 6D**).

### Effect of substitutions on the prognostic power of HTICS Core

**Fig 7:**
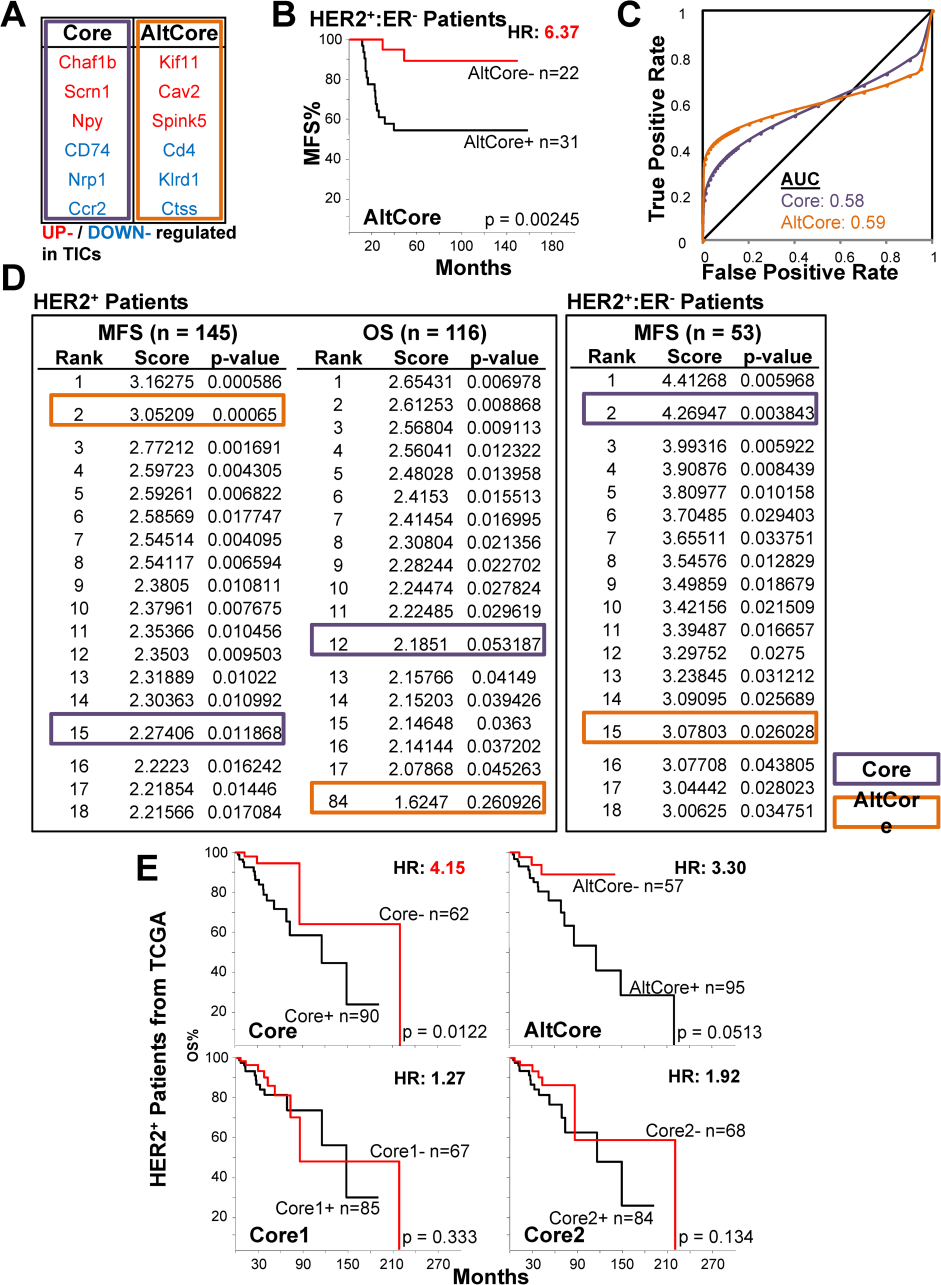
Analysis of HTICS Core. **(A)** AltCore with all 6 Core gene substitutions. (B) Kaplan-Meier analysis of AltCore on MFS cohorts. (C) ROC analysis of AltCore. (D) Core and AltCore tested against 1000 randomly generated signatures of equal numbers of up- and down-regulated genes. (E) Kaplan-Meier OS analyses of Core, AltCore, Core1 and Core2 on HER2^+^ breast cancer samples with RNA-Seq data (TCGA).

We next examined the ability of the replacement genes to substitute for the Core. By substituting all 6 genes in the Core (**Fig 7A**), the resulting AltCore produced better HR of 6.37 than the Core in OS analysis for HER2^+^:ERα^-^ patients (**Fig 7B**). However, like the Core, AltCore had a low AUC of 0.59 (**Fig 7C**). Comparing the Core and AltCore with 1000 randomly selected sets of 6-genes (with 3 gene-up; 3 gene-down), the Core produced consistently significant result (ranked #15 for MFS and #12 for OS for HER2^+^ patients; #2 for MFS in HER2^+^:ERα^-^ patients), whereas AltCore was very unstable ranking #2 for MFS but #84 for OS (**Fig 7D**). Therefore, while the substitutes were able to provide prognostication, they are less stable in predicting patient outcome than the original HTICS or optimized Core.

### Cross-platform analysis of the HTICS Core

Finally, to validate the efficacy of the Core, we analyzed RNA-Sequencing profiles of invasive HER2^+^ breast cancer patients with OS data from The Cancer Genome Atlas (TCGA) [2]. Survival analysis demonstrated that HTICS Core significantly predicted OS with HR of 4.15 (**Fig 7E**). The AltCore, Core1 and Core2 gave similar trends but none was significant (**Fig 7E**). Therefore the 6 gene Core identified here was consistent in making prognostic predictions on independent datasets generated by different technologies (RNA microarray and RNA-seq).

### Comparison of HTICS and Core signatures by empirical false detection rate

**Fig 8:**
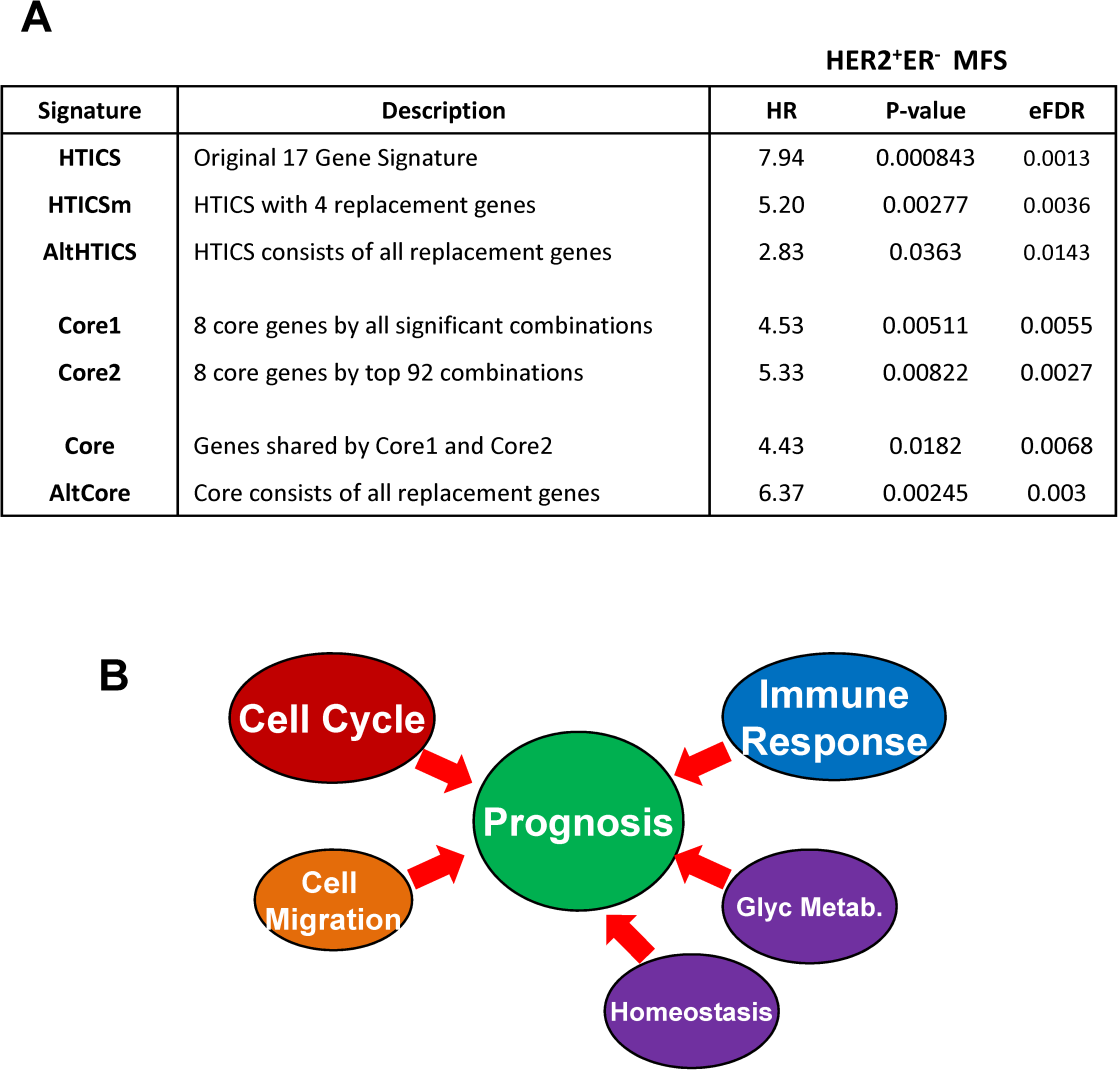
A Model for signaling pathways that drive the prognostic power of HTICS. (A) Assessment of HTICS and derived signatures by hazard ratio, significance and Empirical False Detection Rate (eFDR) analysis. (B) A schematic model for pathways that drive the prognostic power of HTICS. Our analysis reveals that cell cycle and immune response pathways as well as cell migration are critical components, but that additional pathways—homeostasis and Glycan metabolism—also contribute to the full prognostic power of HTICS.

To compare all the signatures described herein we used Empirical False Detection Rate (eFDR). This was calculated by 10,000 permutations using the entire microarray dataset. Each permutation generated a randomly selected gene sets for HR calculation and the rate of random gene sets with significantly better HR than HTICS was recorded (hence eFDR). After 10,000 permutations, only 13 random gene sets had better HR than the original signature and thus, HTICS had eFDR of 0.00129 (**Fig 8A**). All six other signatures developed herein were analyzed in a similar way. The results clearly show that while all core signatures were prognostic, significant and with low eFDR, HTICS was by far the most powerful by all criteria. Thus, while the various pathways, in particularly Cell Cycle, Immune Response and Cell Migration, were sufficient to predict clinical outcome, the other pathways (Homeostasis and Glycan Metabolism) fine-tuned the signature and maximized its prognostic power (**Fig 8B**).

## Discussion

We report the identification of critical pathways mediating the prognostic power of HTICS, a 17-gene signature for HER2^+^:ERα^-^ breast cancer [23]. We identified cell proliferation, cell migration and immune response pathways as mots critical. Two other pathways, homeostasis and Glycan Metabolism, albeit not as important, contributed to optimal prognostication. We found that 15 of 17 (88%) substituting genes that could best replace the 17 HTICS genes were from the same biological pathways, highlighting their importance in predicting disease-free and overall survival for these patients. We also identified a core signature of 6 genes with high prognostic power and showed that they also include genes from the three major pathways. Thus, not all genes in HTICS equally contribute to its prognostic power. Our results suggest that IHC-based detection of the three major pathways—cell proliferation, cell migration and immune response—may successfully predict clinical outcome for HER2^+^:ERα^-^ breast cancer patients. Indeed, we are currently performing retrospective analysis on large cohorts of HER2^+^:ERα^-^ patients using the NanoString assay developed herein. In addition, drugs/inhibitors that can antagonize the Core or substituting genes identified herein may have therapeutic benefits.

Our finding of immune-response gene pathway in HTICS, especially of those associated exclusively with PBMC, seems counterintuitive given that the signature was derived from mouse MMTV-Her2/Neu tumor epithelial cells that were depleted of other cell lineages (endothelial and hematopoietic cells[23]). However, the non-TIC fraction was evidently contaminated with immune-cells, possibly due to incomplete lineage depletion or to lack of expression of cell surface markers used for lineage depletion (CD31; CD45-TER119, respectively). In hindsight, the inclusion of these cells, reflecting normal anti-cancer responses by the host, allowed us to generate a signature that includes immune-response genes that is critically important for the prognostic power of HTICS. Indeed, a meta analysis revealed that immune-response is an important determinant in the prognosis of HER2^+^ breast cancer relative to other subtypes [28]. Notably, the identification of immune-related genes as part of HTICS was only made possible because we identified MMTV-Her2/Neu TICs in isogenic (FvB) mice with intact immune system. Had we attempted to generate HTICS from human HER2^+^ TICs injected into immunocompromised mice, we would have not had the immune component of HTICs, and thus, not a strong prognostic signature. As noted, the presence of immune response pathway even in the minimal 6-gene Core, is particularly interesting given the effect of the immune system on the therapeutic effects of trastuzumab [31]. Combination therapy with trastuzumab and immunomodulatory drugs may therefore be highly synergistic for HER2^+^:ERα^-^ patients.

One important question is whether the HTICS or Core signature genes identified here merely monitor aggressive behaviour of HER2^+^:ERα^-^ breast cancer, or whether they also actively participate in and drive metastatic growth. The identification of a 6 Core gene set as well as substitutes may simplify this question by focusing on the most critical genes. For example, inhibition/knockdown of the only Core cell cycle gene Chaf1b, (Chromatin assembly factor 1, subunit B), which is required for assembly of histone octamers onto newly-replicated DNA, and/or its substitutes Kif11 (Kinesin Family Member 11), which is required for chromosome positioning, centrosome separation and bipolar spindle formation during cell mitosis, may diminish proliferation or self-renewal of HER2^+^:ERα^-^ TICs.

Over-expression of Nrp1 (Neuropilin 1), a vascular endothelial cell growth factor (VEGF) 165 Receptor, has been implicated in the vascularization and metastasis of many cancer types, including breast cancer [37]. Our results suggest that anti-NRP1 therapy such as the synthetic peptide, EG3287, [38], may be assessed against HER2^+^:ERα^-^ breast cancer. The Nrp1 substitute, Klrd1 (Killer Cell Lectin-Like Receptor Subfamily D, Member 1), an antigen expressed on Natural Killer cells, may also be considered as target to block tumor dissemination. Finally, as noted, the homeostasis gene, Atp7b is required for efflux of copper out of the cells. Interestingly, disulfiram, a potent anti-cancer agent, kills tumor cells by increasing intracellular copper concentrations [39, 40].

The identification of cell proliferation, migration and immune-response pathways as key prognostic determinants in HER2^+^:ERα^-^ breast cancer suggests that IHC assays that can faithfully detect alterations in these pathways may be highly prognostic. While proliferation and immune-response can readily be tested by IHC, identification of cell migration markers may be more challenging. A starting point may be the HTICS Cell migration pathway genes (Nrp1, Npy, Cldn8) and their substitutes (Klrd1, Spink5, Itga8) identified herein—but other known markers of cancer cell dissemination or circulating tumor cells may be equally informative [41, 42].

## Conclusion

In conclusion, using bioinformatic analysis we established a rationale for identifying critical pathways as well as substitute genes and Core Signature genes that would facilitate clinical application of HTICS and may uncover new therapeutic targets for HER2^+^:ERα^-^ breast cancer. Our analysis should be applicable to other types of multigene-based prognostic signatures for breast and other cancer types, and may facilitate the development of a universal breast cancer prognostic test for multiple breast cancer subtypes.

## Supporting information

S1 Fig ROC analysis comparing HER2 status as determined by amplicon genes vs IHC, and comparing the prognostic power of HTICS in HER2^+^:ERα^-^ vs HER2^+^:ERα^+^ patients. (A) ROC analysis comparing HER2 status as determined by amplicon genes vs IHC. (B) ROC analysis demonstrating that HTICS is most effective in prognostication of HER2^+^:ERα^-^ patients. (C) Heatmap for HTICS gene expression in HER2+ patients with corresponding SSM scores and incidents of metastasis.
(TIF)

S2 Fig Effect of substituting other-pathway genes for AltHTICS. Using the highest correlated genes from different pathways instead of genes in the same pathway (Rnase6 for CCR2 and Mphosph6 for Scrn1) (A), the prognostic ability of the signatures: Rnase6 in AltHTICSb and Mphosph6 in AltHTICSc, were compared with the original AltHTICS by Kaplan-Meier analysis of HER2^+^:ERα^-^ patients with MFS data (B).
(TIF)

S3 Fig Effect of removing individual functional pathway on the prognostic power of HTICS. (A) Kaplan-Meier analysis of HER2^+^:ERα^-^ patients with MFS data in leave-one-out analysis of the HTICS pathways. (B) ROC analysis for HER2^+^:ERα^-^ patients with MFS data in leave-one-out analysis of the HTICS pathways.
(TIF)

S1 Table Datasets and samples with microarray and HER2 IHC for 5-gene amplicon assessments. (XLSX)

S2 Table Overall and metastasis-free survival datasets with no herceptin treatment and having all breast cancer subtypes represented. (XLSX)

S3 Table Metastasis-free survival incidences and time for all samples. (XLSX)

S4 Table Nanostring counts for HTICS, backup, and control genes in a panel of FFPE breast cancer samples. (XLSX)
