# Supplementary figures and images for: Identification of cell proliferation, immune response and cell migration as critical pathways in a prognostic signature for HER2+:ERα- breast cancer
Source: PLoS One. 2017 Jun 20;12(6):e0179223. doi: 10.1371/journal.pone.0179223 (PMC5478114; doi:10.1371/journal.pone.0179223)

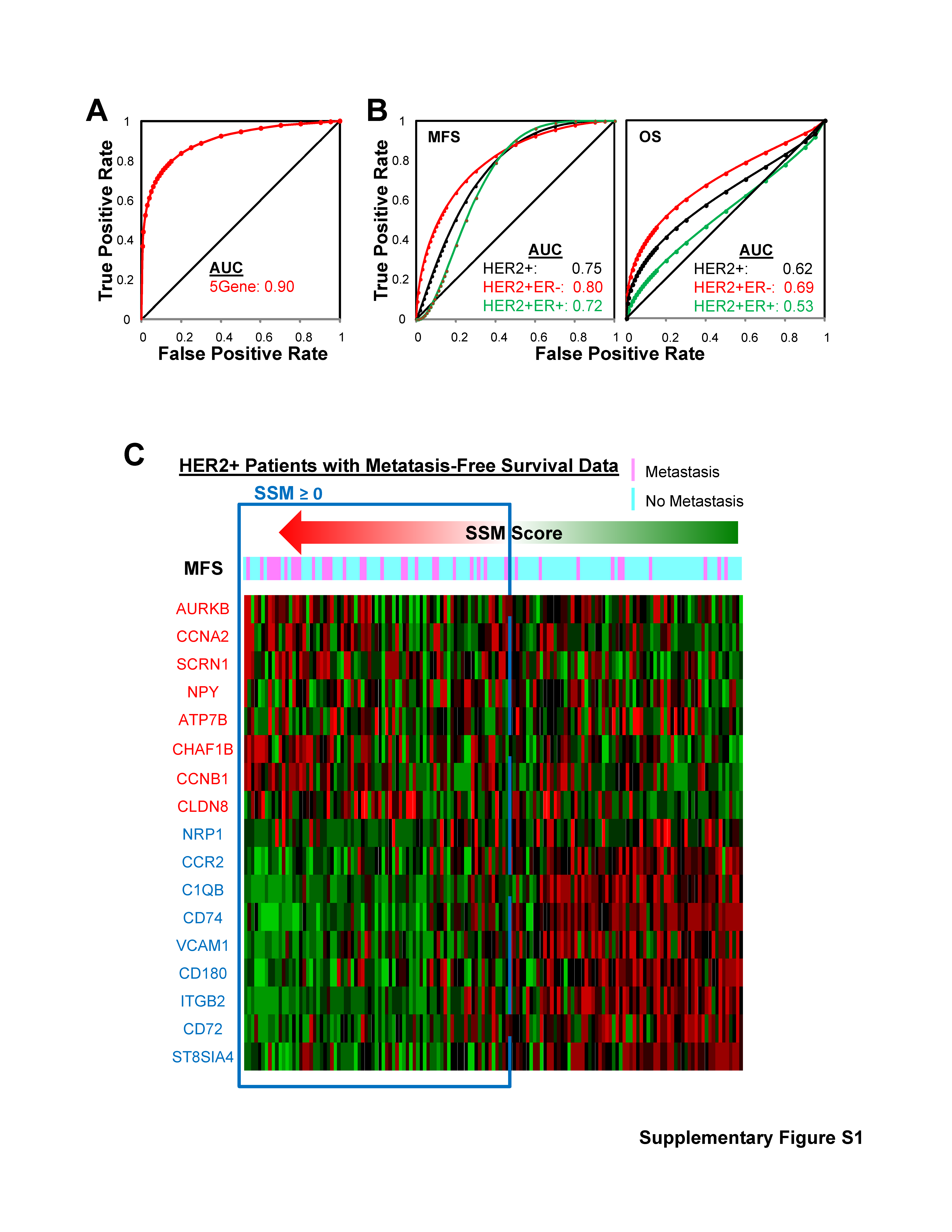

Supplement: S1 Fig — (A) ROC analysis comparing HER2 status as determined by amplicon genes vs IHC. (B) ROC analysis demonstrating that HTICS is most effective in prognostication of HER2+:ERα- patients. (C) Heatmap for HTICS gene expression in HER2+ patients with corresponding SSM scores and incidents of metastasis. (TIF) [file pone.0179223.s001.tif]

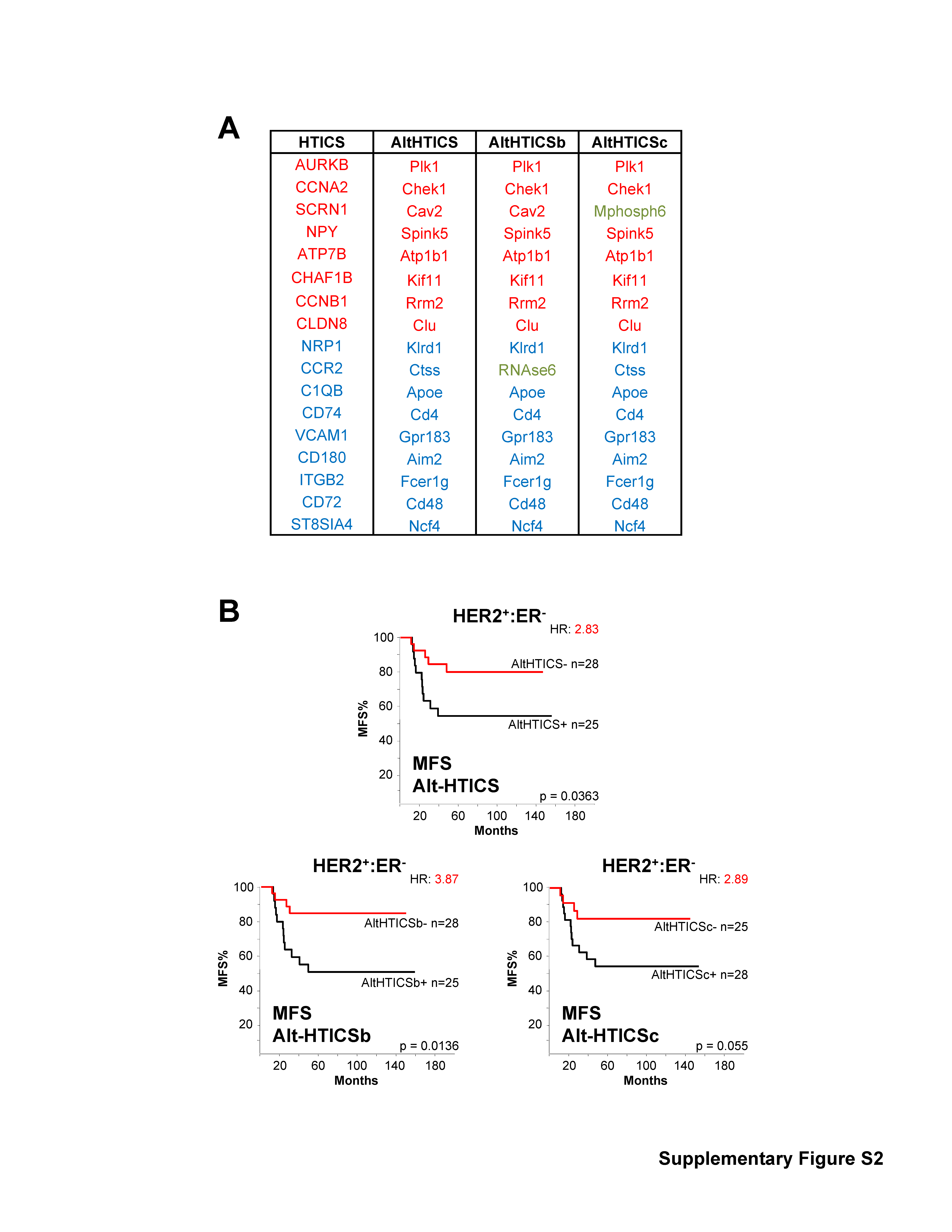

Supplement: S2 Fig — Using the highest correlated genes from different pathways instead of genes in the same pathway (Rnase6 for CCR2 and Mphosph6 for Scrn1) (A), the prognostic ability of the signatures: Rnase6 in AltHTICSb and Mphosph6 in AltHTICSc, were compared with the original AltHTICS by Kaplan-Meier analysis of HER2+:ERα- patients with MFS data (B). (TIF) [file pone.0179223.s002.tif]

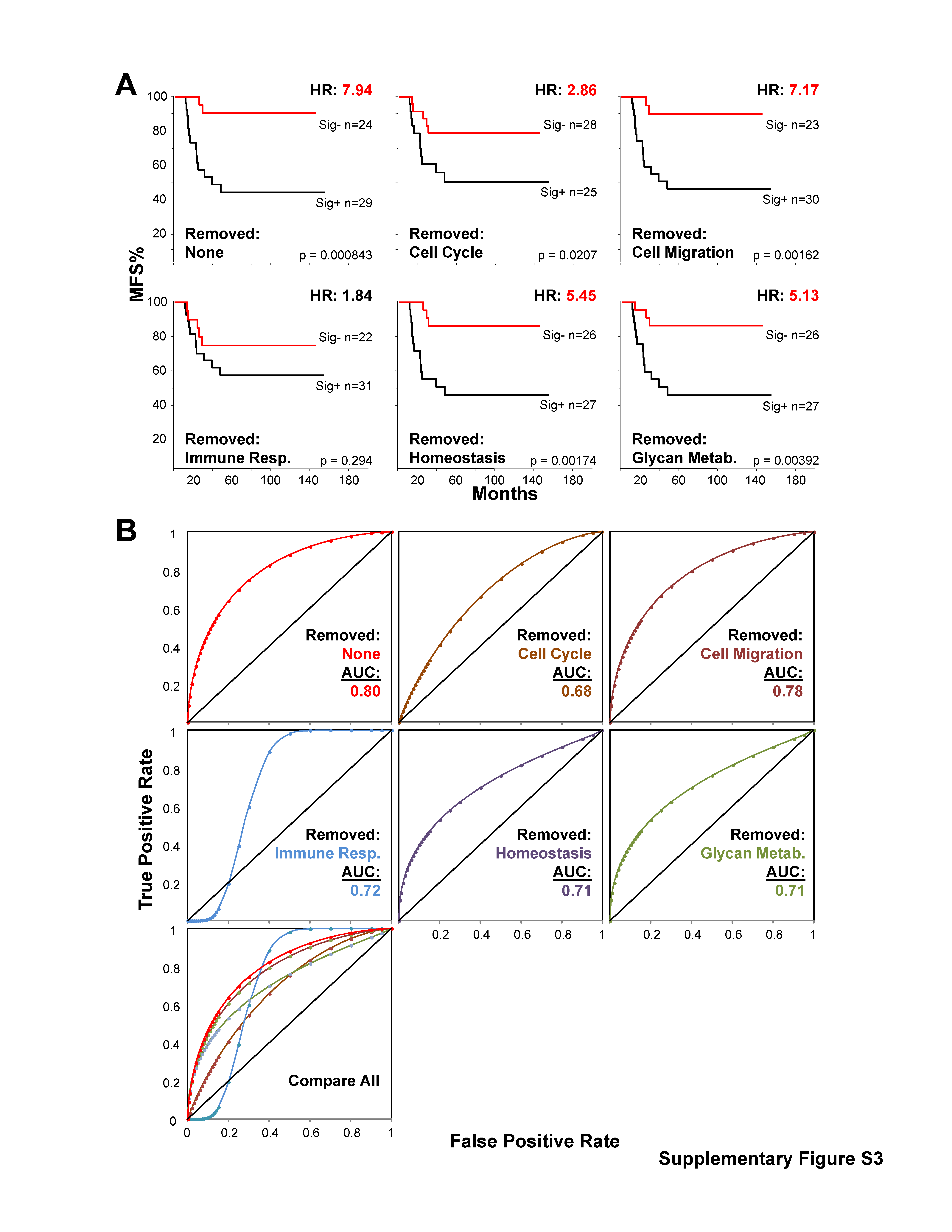

Supplement: S3 Fig — (A) Kaplan-Meier analysis of HER2+:ERα- patients with MFS data in leave-one-out analysis of the HTICS pathways. (B) ROC analysis for HER2+:ERα- patients with MFS data in leave-one-out analysis of the HTICS pathways. (TIF) [file pone.0179223.s003.tif]
